# A ‘Grantathon’ model to mentor new investigators in mental health research

**DOI:** 10.1186/s12961-017-0254-0

**Published:** 2017-10-24

**Authors:** Mary Hawk, Vishwajit Nimgaonkar, Triptish Bhatia, Jaspreet S. Brar, Wafaa Abdelhakim Elbahaey, James E. Egan, Prasad Konasale, Supriya Kumar, Margaret C. McDonald, Ravinder Singh, Soumya Swaminathan, Joel Wood, Smita N. Deshpande

**Affiliations:** 1 Behavioral and Community Health Sciences, Evaluation Institute for Public Health, University of Pittsburgh, Graduate School of Public Health, 4136 Parran Hall - 130 DeSoto Street, Pittsburgh, PA 15261 United States of America; 2 Psychiatry and Human Genetics, University of Pittsburgh School of Medicine and Graduate School of Public Health, Pittsburgh, PA United States of America; 3 Program for Genetics and Psychosis, University of Pittsburgh School of Medicine and Graduate School of Public Health, Pittsburgh, PA United States of America; 4 Indo-US Projects, Department of Psychiatry, Centre of Excellence in Mental Health, PGIMER-Dr. Ram Manohar Lohia Hospital, Bangabandhu Sheikh Mujib Road, New Delhi, 110001 India; 5 Department of Psychiatry, Western Psychiatric Institute and Clinic, Community Care Behavioral Health Organization, University of Pittsburgh School of Medicine, Pittsburgh, PA 15213 United States of America; 6 Psychiatry, Faculty of Medicine, Mansoura University, Mansoura, Egypt; 7 Research and Graduate Affairs, Faculty of Medicine, Mansoura University, Mansoura, Egypt; 8 Behavioral and Community Health Sciences, Graduate School of Public Health, University of Pittsburgh, Pittsburgh, PA United States of America; 9 Psychiatry, University of Pittsburgh School of Medicine, Pittsburgh, PA United States of America; 10 Academic Affairs, Health Sciences, University of Pittsburgh, Pittsburgh, PA United States of America; 11 Epidemiology, Graduate School of Public Health, University of Pittsburgh, Pittsburgh, PA United States of America; 12 Psychiatry, School of Medicine, University of Pittsburgh, Pittsburgh, PA United States of America; 13 Scientist C, Indian Council for Medical Research, New Delhi, India; 14 Department of Health Research, Ansari Nagar, New Delhi, India; 15 Director General, Indian Council of Medical Research, Ansari Nagar, New Delhi, India; 16 Department of Psychiatry, University of Pittsburgh School of Medicine, Pittsburgh, PA 15213 United States of America; 17 Department of Psychiatry, Centre of Excellence in Mental Health, PGIMER-Dr. Ram Manohar Lohia Hospital, Bangabandhu Sheikh Mujib Road, New Delhi, 110001 India

**Keywords:** Mental health research, Mental health treatment gap, Capacity-building, Low and middle-income countries, India

## Abstract

**Background:**

There is a critical gap between needs and available resources for mental health treatment across the world, particularly in low- and middle-income countries (LMICs). In countries committed to increasing resources to address these needs it is important to conduct research, not only to assess the depth of mental health needs and the current provision of public and private mental health services, but also to examine implementation methods and evaluate mental health approaches to determine which methods are most effective in local contexts. However, research resources in many LMICs are inadequate, largely because conventional research training is time-consuming and expensive. Adapting a hackathon model may be a feasible method of increasing capacity for mental health services research in resource-poor countries.

**Methods:**

To explore the feasibility of this approach, we developed a ‘grantathon’, i.e. a research training workshop, to build capacity among new investigators on implementation research of Indian government-funded mental health programmes, which was based on a need expressed by government agencies. The workshop was conducted in Delhi, India, and brought together junior faculty members working in mental health services settings throughout the country, experienced international behavioural health researchers and representatives of the Indian Council for Medical Research (ICMR), the prime Indian medical research funding agency. Pre- and post-assessments were used to capture changes in participants’ perceived abilities to develop proposals, design research studies, evaluate outcomes and develop collaborations with ICMR and other researchers. Process measures were used to track the number of single-or multi-site proposals that were generated and funded.

**Results:**

Participants (n = 24) generated 12 single- or multi-site research grant applications that will be funded by ICMR.

**Conclusion:**

The grantathon model described herein can be modified to build mental health services research capacity in other contexts. Given that this workshop not only was conceptualised and delivered but also returned results in less than 1 year, this model has the potential to quickly build research capacity and ultimately reduce the mental health treatment gap in resource-limited settings.

## Background

Mental health and substance use disorders comprise 7.4% of global disability-adjusted life years and 22.7% of global years lived with disability [1]. Much of the disability occurs in low- and middle-income countries (LMICs) such as India, where individuals face the dual challenges of limited healthcare provision and the stigma and heavy economic burden of mental illness. This combined burden often traps individuals in a downward spiral and, in turn, increases the mental health treatment gap [2]. To address these deficiencies, WHO, in its Global Action Plan for the Prevention and Control of Noncommunicable Diseases 2013–2020, proposed a challenging agenda for change, mapping a plan to strengthen mental health leadership and policymaking, provide comprehensive and integrated community-based care, implement prevention strategies and strengthen information systems to support data-driven decision-making [3].

Building capacity for health research in developing countries is integral to the goals of building effective interventions and strengthening existing infrastructure. Bolstering mental health services research capacity will enable LMICs to pinpoint interventions that are effective in their cultural and geographic settings, to apply and replicate results, and ultimately to strengthen mental health service systems. Unfortunately, in many LMICs, including India, mental health research has lagged [4]. In addition, there is a relatively low proportion of Indian research publications in international journals, which demonstrates lost opportunities to disseminate knowledge regarding effective approaches [5]. In India, structural inequalities persist, creating funding and methodological challenges that differ by region, making national research collaborations and capacity-building efforts difficult to implement and sustain.

One way to address these gaps is to increase research capacity in medical colleges in India [6]. Indian medical education has historically been divorced from research, in part due to significant shortages in funding and in faculty members who can provide research training. The Medical Council of India mandates that medical students complete a thesis based on practical research. However, these studies are rarely published, suggesting missed opportunities to disseminate findings, create research collaboratives and apply research findings at the regional or national levels. Bridging this gap and ensuring sustainable research are serious challenges, since research training is traditionally intensive, time consuming and expensive. Because there is a paucity of human resources even for delivering clinical care, formal research training is viewed as an unaffordable luxury in LMICs.

India accounts for 15% of the disease burden of global psychiatric, neurological and substance use disorders, which is greater than that in all LMICs combined. Depressive and anxiety disorders are the most common mental illnesses in India [7]. According to the Summary document of the National Mental Health Survey 2016 [7], 10.6% of the population suffers from any mental health or substance use disorder. Alcohol and tobacco use disorders were the most common, followed by neurotic and stress-related disorders. The burden of these disorders in India is projected to increase by 23% between 2013 and 2025 [7]. Unfortunately, only approximately 10% of those living with psychiatric disorders in India are thought to receive evidence-based care [8]. More than 60% of people with mental disorders receive care in district hospitals, but up to 40% of patients must travel 10 km or more to access services [9].

To address its unmet mental health needs, India initiated a National Mental Health Programme (NMHP) in 1982, followed by the launch of the District Mental Health Programme (DMHP) in 1996. The goal of the DMHP is to provide community-oriented services to reduce the burden and impact of mental health disorders. As of April 2015, the DMHP covered 36% of all districts in the country [10]. However, a significant mental health treatment gap persists due to restricted funding and shortages of human resources. Even in many districts where the DMHP operates, services are limited to mental health outreach clinics in a few primary healthcare centres. In addition, systematic evaluation of the efficacy of the DMHP is an ongoing challenge.

In recognition of these issues, in May 2016, the Indian Council of Medical Research (ICMR) organised a meeting of experts entitled Brainstorming on Prioritization of Mental Health Research in India (http://www.icmr.nic.in/final/Report_Brainstorming.pdf). The goal of the meeting was to identify priority areas for Indian research on mental health and substance use and misuse for the next 5 years. One of the priorities that evolved from the meeting was to initiate training to build research capacity among medical and paramedical staff throughout India to address the mental health services gap. The experts acknowledged that, in view of resource limitations, formal research training for even a small number of individuals could be challenging. An advisory committee was created, which held a series of in-depth discussions regarding directions for research capacity-building. The advisory committee suggested a workshop model to identify, motivate and mentor promising individuals in conducting mental health research. In addition to skills development, the workshop would provide the opportunity for participants to develop research funding proposals, which would be submitted directly to ICMR at the conclusion of the workshop. ICMR agreed to review these proposals and consider funding those deemed worthy of support. Thus, the workshop was innovative in its plan to not only build participants’ skills, but also to provide a direct interface between two critical groups, namely clinicians providing and evaluating mental health services and ICMR leadership, who had the authority to prioritise the country’s mental health needs and make funding decisions to address those needs. Through the workshop, it was hoped that individualised mentorship would not only enable trainees to acquire research planning skills but also to establish links between the trainees, their mentors and ICMR leadership. In this way, workshop participants would contribute to evaluation research for the ICMR-sponsored mental health services research in an efficient and productive manner.

This paper describes the development and implementation of a ‘grantathon’ workshop model to address the mental health services research gap by building capacity among junior and mid-level faculty members providing mental health services in clinic- or community-based settings throughout India. We describe the background and development of the capacity-building workshop loosely based on a ‘hackathon’ model, which is a form of crowd sourcing intended to spur innovation among groups with diverse backgrounds that learn from each other and work collaboratively toward a common goal. We also describe our methods of evaluation and data collection, workshop outcomes and planned next steps.

## Methods

### Programme development

During the priority-setting meeting in which the idea was conceived, ICMR asked two researchers, one from the University of Pittsburgh and another from the Post Graduate Institute of Medical Education and Research at Dr. Ram Manohar Lohia Hospital (PGIMER-Dr. RML), to spearhead a workshop to improve mental health services research capacity in India. These individuals have a long history of collaboration in research infrastructure building and research training programmes in India and Egypt, funded by the Fogarty International Center, National Institutes of Health, United States of America.

A plan to develop the Capacity Building Workshop on Implementation Research under the NMHP was initiated. Fifteen faculty members with collective expertise exceeding 300 years of experience in mental health intervention research were invited to join the workshop planning committee alongside officials from the ICMR. The faculty members were recruited from the University of Pittsburgh, University of Mansoura, Egypt, the Indian Schizophrenia Research Foundation, and the PGIMER-Dr. RMLH in New Delhi, India.

In July 2016, the ICMR programme directors and the invited faculty members conducted a series of telephone-based planning meetings. Approximately 1500 collective hours of work, or an average of 100 hours per expert, were expended on workshop development. Topic areas identified for inclusion in the workshop were programme development and evaluation, study design, data management, proposal writing, bioethics and health equity. By the end of the workshop, each participant was expected to have developed a collaborative or single-investigator funding proposal for submission to ICMR. To address the ultimate goal of improving the mental health services gap, the workshop objectives were to build intervention, evaluation and mental health services research capacity among junior/mid-level faculty members as well as to facilitate collaborative efforts among participants, create mentoring partnerships with faculty members, and strengthen networking among ICMR and researchers throughout India.

### Participant recruitment and selection

In addition to planning the workshop curriculum (Table 1), a process for recruiting and screening potential participants was developed, which required interested individuals to apply and be accepted to the programme. ICMR advertised the workshop plan on its website, and several mental health professional e-groups were informed of the opportunity. Repeat advertisements were sent to some institutions with fewer resources or in remote regions of India. Applications were also invited directly from research faculty below the age of 45, since the goal was to build capacity among early stage investigators who were more likely to have many more years ahead of them working in the field. Individuals with faculty appointments in medical colleges and non-governmental organisations involved in mental health research were also prioritised. Applicants were required to submit curricula vitae as well as letters of support from the directors of their institutions assuring sufficient time and resources to implement the projects should they be funded. Each applicant was also asked to provide a brief outline of a project or intervention of interest and a description of a target population that was consistent with any of the ICMR priority areas, namely NMHP, DMHP, depression (especially post-partum or associated with communicable/non-communicable disease), suicide prevention and tribal health. There was no requirement for prior research funding.

**Table 1:** Workshop agenda

Session	Session time	Topic/objectives
1	Day One AM	Inauguration: Indian Council of Medical Research
2	Day One AM-PM	Completion of pre-workshop questionnaire, discussion of expectations; participant introductions and presentation of proposed projects
	Day One PM	Post-meeting discussions and reclassification (faculty)
3	Day Two AM	Recap and review of previous day’s work; health equity; grant writing (critiquing an idea); common study designs; ICMR: successful project writing, budget and plan for projects
4	Day Two PM	Practice session: participants develop evaluation questions to be addressed through their proposed studies
5	Day Three AM	Recap and review of previous day’s work Evaluation framework
6	Day Three PM	Practice session: proposal writing
7	Day Four AM	Recap and review of previous day’s work Critical appraisal of research data management
8	Day Four PM	Practice session: proposal writing
9	Day Five AM	Post workshop evaluation
10	Day Five PM	Presentation of workgroup proposals
11	Day Five PM	Reporting out and next steps; budget development and planning; workshop evaluation and feedback-long term follow-up by mentors

Members of the workshop planning group and the ICMR reviewed and selected workshop participants. Applications were reviewed for appropriateness of research topic, applicants’ experience and interest, and applicability to NMHP. Fifty applications were received from all over India, from which 25 individuals were selected in order to provide highly individualised training. To maximise the learning opportunities, invitees were provided with advance reading materials and video lectures addressing logic models and various aspects of research methodology.

### Programme implementation

Mentors with expertise in relevant areas of research were identified at the University of Pittsburgh, Mansoura University, and PGIMER-Dr. RML Hospital to provide workshop training. Their expertise included bioethics, evaluation and intervention research, health services research, grant writing, statistics, psychiatric epidemiology and phenomenology, and healthcare delivery.

The Capacity Building Workshop on Implementation Research under the NMHP took place over 5 days in November 2016. The training took place at the National Institute of Health and Family Welfare, Munirka, New Delhi, and was provided at no cost to participants or their sponsoring agencies. Twenty-four of the 25 invited applicants participated alongside 14 mentors. On acceptance of their applications for the workshop, phone, e-mail, and an international messaging app were used to connect workshop participants with each other and with mentors even before the workshop took place.

On each day of the workshop, faculty members delivered lectures in the morning, followed by team-based proposal development each afternoon to enhance the adoption of skills (Table 1). To facilitate collaboration, participants were divided into groups based on topic area or research approach (Table 2). Each group included two or more mentors with relevant expertise. In addition, breakout groups served to pair participants with faculty members, with the aim of establishing international mentoring relationships. Participants were also provided with ‘homework’ assignments overnight such as revising/strengthening proposals, developing supplementary materials for their own research proposals or obtaining relevant references. Time was also built in to each day for presentation, discussion and peer-review of progress. As most of the workshop participants stayed on site, extensive networking opportunities were available throughout the workshop. In the concluding session of the workshop, draft proposals were presented to the ICMR Director General and the Joint Secretary of the NMHP, who discussed the subsequent steps in mental health services research. Thus, participants received direct, face-to-face feedback about their proposals in the context of ICMR funding priorities.

**Table 2:** Geographic region, foci, and multi- versus single investigator status of developed projects

Geographic region	Final proposal	Multi- vs. single investigator
Kashmir (North India)	Community-based intervention on mental health in Kashmir	Single investigator
Karnataka (South India)	Implementation and evaluation of the NIMHANS-ECHO blended training program for the district mental health programme (DMHP) workforce in a rural south-Indian district of Karnataka state	Multi-center
Kerala (South India)	Evaluation of DMHP psychiatric services to the severely mentally ill in their old age	Single investigator
Jharkhand (East India)	Outcome of services at the community extension clinics for patients with common mental disorders: a client-centred approach	Single investigator
Tamil Nadu (South India)	Development of a community level module for physical illnesses in patients with psychiatric illness	Single investigator
Karnataka (South India)	Effectiveness of community based rehabilitation delivered by accredited social health activists for persons with severe mental illness in a rural community in Karnataka: a randomised controlled comparison with specialist-delivered care	Single investigator
New Delhi	Development and validation of the screening version of Indian scale for assessment of autism	Single investigator
Karnataka (South India), New Delhi (North India)	A multi-centric randomised controlled trial to evaluate the efficacy of telephone-based psychosocial interventions on future suicide risk in suicide attempters	Multi-center
Karnataka (South India)	Psychological intervention by videoconference for vulnerable family members of farmers who have committed suicide	Single investigator
Karnataka (South India), New Delhi (North India), Maharashtra (West India), Karnataka (South India)	A multi-centric randomised controlled trial to assess the effectiveness of screening and a brief nurse-delivered intervention for depression in pregnancy	Multi-center
New Delhi (North India), Karnataka (South India)	Managing depression in diabetes: a multi-centre randomised controlled efficacy trial comparing fluoxetine and mindfulness in primary care setting	Multi-center
Mizoram (North East India), Karnataka (South India), Gujarat (West India)	Alcohol use among adolescent tribals in three corners of India: prevalence and pilot intervention studies	Multi-center

ICMR leaders were present throughout the workshop, enabling them to amplify and clarify NMHP research goals and priorities for the participants in real time. For example, the ICMR the Director General, Additional Director General, Chief of Non-Communicable Diseases, and Senior Director of Finance shared their vision for improving mental health outcomes in India on the first day. The Senior Scientist for Mental Health in ICMR was present along with his team throughout the workshop. During the summing up session, the Director General, Chief of Non-Communicable Diseases, and Senior Scientist (Mental Health) were present, along with the Joint Secretary (NHMP) of the Central Ministry of Health and Family Welfare, to review all proposals.

### Programme evaluation

The outcomes of the workshop were evaluated at several levels, listed below, and independent national experts were also consulted for their assessments of the workshop.

#### Changes in participants’ perceived ability to conduct mental health services research

Participants were asked to complete a 9-item self-assessment questionnaire at the beginning and end of the capacity-building workshop, addressing perceived ability to develop proposals, design research studies, evaluate outcomes and develop collaborations with ICMR and other researchers (Tables 3 and 4). Paired samples t-tests were conducted in SPSS to assess for significant differences in pre- and post-test scores.

**Table 3:** Participant self-assessment measures and results

*Likert scale (1–5)* *1 = Strongly agree and 5 = Strongly disagree*			
	Pre-test mean (SD)	Post-test mean (SD)	Paired sample t-test *P* value*
1. I have confidence in my proposal-writing skills	2.62 (0.77)	1.58 (0.58)	<0.05*
2. I can design an intervention that is likely to achieve its goals	2.29 (0.46)	1.58 (0.58)	<0.05*
3. I can develop a study design that will help me to know if my intervention achieved its desired outcomes	2.50 (0.66)	1.58 (0.58)	<0.05*
4. I can develop evaluation methods that will help me to know if my intervention achieved its desired outcomes	2.75 (0.74)	1.75 (0.74)	<0.05*
5. I can create a strong funding proposal	3.08 (0.88)	1.88 (0.68)	<0.05*
6. I can develop a mentoring relationship that I believe will help me to continue the learning process	1.96 (0.77)	1.39 (0.58)	0.012*
7. I can develop collaborative relationships with other participants from within my own institute that I plan to continue	1.75 (0.79)	1.42 (0.78)	0.175
8. I can develop collaborative relationships with other participants from outside my own institute that I plan to continue	2.00 (0.89)	1.50 (0.66)	0.049*
9. I can expand relationships with ICMR faculty to advance my work in this field	2.04 (0.75)	1.33 (0.48)	0.001*
Sum of all questions	21.22 (4.37)	14.17 (3.54)	<0.05*

*Significant at the 0.05 probability level

**Table 4:** Raw data: participant self-assessment measures and results

Participant ID	Pre1	Pre2	Pre3	Pre4	Pre5	Pre6	Pre7	Pre8	Pre9	Post1	Post2	Post3	Post4	Post5	Post6	Post7	Post8	Post9
1	2	3	3	2	2	1	2	2	2	2	2	1	2	3	1	1	1	1
2	4	2	3	4	4	2	2	3	3	1	1	1	1	1	1	1	1	1
3	3	2	2	3	4	3	2	2	2	2	2	2	2	3	1	1	1	1
4	3	2	4	4	4	2	2	2	2	2	3	2	2	3	3	2	3	2
5	4	2	2	3	3	1	1	1	1	1	1	2	1	2	1	1	2	1
6	2	2	2	3	2	2	2	2	2	3	2	2	3	1	2	1	1	1
7	3	2	2	2	3	1	1	1	1	2	2	2	2	2	2	1	1	2
8	4	3	3	3	4	3	4	4	2	1	2	1	1	2	1	1	1	2
9	2	2	2	2	1	1	1	1	1	2	2	3	4	2	2	3	3	2
10	2	2	3	2	3	2	2	2	2	1	1	2	1	1	1	1	1	1
11	2	2	2	3	3	2	2	2	2	2	2	1	1	3	1	1	1	1
12	4	3	4	4	4	1	1	2	3	2	2	2	2	2	2	2	2	2
13	2	2	2	2	2	2	2	2	2	1	1	2	2	1	1	1	1	1
14	2	2	2	3	3	1	1	1	1	2	2	1	1	2	1	1	1	1
15	3	3	3	4	2	2	1	2	2	1	2	2	2	2	1	2	2	2
16	2	3	3	3	4	3	3	3	3	2	2	2	2	1	1	2	2	1
17	3	2	2	2	2	9	1	1	1	1	1	1	1	2	1	1	1	1
18	2	2	2	2	3	3	3	3	3	1	2	1	1	2	2	1	1	1
19	2	2	2	2	3	3	2	3	3	1	1	1	2	2	1	4	1	1
20	3	3	2	3	4	2	2	3	2	1	1	2	2	1	1	1	2	1
21	2	2	2	3	4	2	1	1	2	2	1	1	2	2	2	1	2	1
22	2	2	2	2	3	1	1	1	1	1	1	1	1	1	1	1	1	1
23	3	3	3	3	4	2	2	3	3	2	1	2	2	2	1	1	2	2
24	2	2	3	2	3	3	1	1	3	2	1	1	2	2	2	2	2	2

#### ICMR funding

ICMR created a funding mechanism for participants to submit proposals developed during the workshop. The deadline for submission was 30 days after the workshop ended.

#### Participant collaboration

The workshop faculty members tracked the number of participants who collaborated with them to submit proposals to ICMR and continued to communicate with them after the workshop.

## Results

**Fig. 1:**
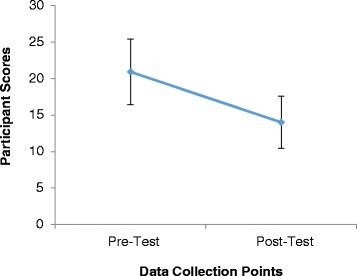
Participants’ pre- and post-workshop self-assessment scores

Workshop participants represented different parts of India and had prior training in psychiatry, psychiatric social work, clinical psychology, social work, pharmacology or community medicine. Though it was not required, two participants had previous funding (from state governments to their institution) and two had received training and research support through NIH-funded training grants to two of the authors (SND and VLN). All trainees had completed research theses or dissertations as a requirement for their post-graduate training. When examining mean differences in pre-test versus post-test self-assessment scores, pairing scores for each participant, we found significant improvements indicating overall gains in each participant’s perceived skills and confidence (t(22) = 5.844, *P* < 0.0001). All but one participant reported improved self-assessment scores at the conclusion of the workshop. Statistically significant improvements for each self-assessment item were noted (Tables 3 and 4), with the exception of responses to the question, “I can develop collaborative relationships with other participants from within my own institute that I plan to continue”. There was a numeric improvement that was not statistically significant for this item. All but one participant noted improvements in perceived skills across all domains in the self-assessment. We also fit a multivariate generalised linear model in SPSS to the data to produce a Hotelling’s trace statistic in order to examine all of the pre- and post-assessment questions as joint outcomes, which was also significant (Hotelling’s T2 = 62.65, f = 5.6, *P* = 0.000072) and further indicates improvements in participants’ self-assessed learning (Fig. 1).

All participants were able to draft full research proposals during the workshop. Participants remained in touch with each other and with their mentors via email and the ‘WhatsApp’ messaging group after the workshop. Within 30 days of workshop completion, all 24 participants successfully submitted a total of 12 individual or multi-centre research proposals to ICMR for funding consideration. Multi-centre proposals were submitted by 17 participants and single centre proposals by the rest (Table 2). Each proposal was critically reviewed by ICMR programme staff and experts, in addition to review by the mentors. Based on these reviews, ICMR decided to fund all the proposals beginning in 2017.

## Discussion

The hackathon model is a form of crowd sourcing intended to spur innovation among groups with diverse backgrounds that learn from each other and work collaboratively toward a common goal. Other features of the hackathon model include focusing on a specific problem, developing a solution using design thinking techniques, pitching the solution to participants, gathering rapid feedback and quickly altering the prototype design. Prior hackathons have focused on stroke innovation, rehabilitation medicine, human cognition and brain mapping, reaching underserved populations in LMICs, and using data for clinical problem-solving.

Our ‘grantathon’ workshop was designed to engage and support new investigators in mental health services research by incorporating elements of the ‘hackathon’ model that has been used to stimulate innovation in health and public health. To our knowledge, this type of process or its evaluation has not been conducted in India or, for that matter, elsewhere in the world. The grantathon model extended the hackathon model by expecting trainees to prepare for the workshop by developing a priori research ideas that they wished to explore and expand into full research proposals. Our workshop design also included didactic sessions and attendees were assigned to several parallel working groups to foster collaboration and team learning. Further, trainees were paired with mentors who led the groups. Like the hackathon model, our approach emphasised sprint-style capacity-building and problem-solving and brought together professionals from several health-related disciplines who could bring their own perspectives to a common problem. The training faculty will continue to provide advice and consultations to the trainees for their funded work pro bono, as required by ICMR regulations, and to facilitate transition of the trainees to independent investigator status.

Participants represented a wide range of mental health-related specialties and were drawn from a mix of primary care facilities, including medical schools and research institutions. The Indian culture is broadly heterogeneous and inclusive of many different languages and religions, and workshop participants represented a wide range of Indian districts from across the country. Participants focused heavily on adaptation and translation of scales and other data collection methods for use in their home regions, and this level of transcultural adaptation improves the likelihood that the programmes designed during the workshop will have high degrees of relevance in the districts in which they will be implemented. The fact that 62.5% of the attendees engaged in collaborative projects, despite representing numerous cultures and geographic regions, demonstrates that the workshop was successful in creating the collegial atmosphere characteristic of the hackathon approach and emphasised by the workshop faculty.

Important communication channels were established between young medical faculty and researchers of international repute, many of them on a one-to-one basis. Participants also created foundations for ongoing collaboration by establishing discussion groups among themselves. Since the emphasis was on cooperation and collaboration, a research model that is less well-developed in India, it is important to note that the majority of workshop participants collaborated with peers and mentors to submit multi-site projects. The key ingredients for the workshop’s success included motivated and focused trainees, dedicated faculty members with complementary skillsets and continued direct involvement by ICMR, the funding agency. We acknowledge that other projects seeking to replicate our model might not have a similar commitment, which could limit their success. We therefore call out to other funding agencies wishing to improve health services in LMICs to consider sponsoring future research learning opportunities that incorporate the funding commitment demonstrated by ICMR in our model.

To spur learning from our capacity-building model, it is important to identify and share, not only the model’s strengths and successes, but also its limitations and opportunities for improvement. One of the challenges in our capacity-building approach was that participants were initially resistant to the idea of submitting collaborative proposals, even though it was one of ICMR’s stated interests. Further, the wide range of research aims presented by the trainees occasionally made it difficult to compose synergistic groups. Strategies used to address these challenges included group discussion among mentors to identify projects and participants that would be well-suited for collaborative work, targeted counselling to specific trainees who had difficulties understanding the concepts, and focused collaborative work between mentors and trainees on the development of succinct research aims. The ICMR was also flexible in accommodating some proposals that were not a primary focus in NMHP, for example, neurodevelopmental disorders and virtual learning.

Only 4 months were available for organising the workshop, posing another hurdle that was addressed by frequent discussions and consensus-based decisions among the training faculty. Because the turnaround time between participants’ application to and acceptance in the workshop was only 1 month, there was also relatively little time available for trainees to complete pre-workshop readings. We recommend that planners begin the workshop development process 12 months in advance of the training opportunity. To enable greater productivity, we also recommend that clear goals be set and key expectations (such as those related to collaboration) be stated explicitly. Further, we recommend a 2-month period prior to the workshop to enable trainees to complete designated reading assignments and to engage with mentors prior to the workshop.

There are several other ways to improve our model. We recommend that attendees be asked to provide detailed information, including background, study design, methods and availability of data about the research question to be addressed during the workshop. We found that many applicants described the problem to be addressed in great detail but had far less to say about study design or methods. By providing more guidance in advance, we could begin ‘pre-mentoring’ before the workshop and offer an application format that would enable workshop planners to evaluate applications equitably. A pre-workshop survey would help planners to identify participants’ learning needs. For example, our attendees’ need for qualitative research methods was not anticipated, possibly because mentors were more accustomed to the need for quantitative research methods training. In the same vein, pairing mentors with participants and establishing communications between participants prior to the workshop could accelerate the rapport-building and learning processes necessary to facilitate research collaborations. In the international arena, country-specific guidelines for peer review would enable participants to tailor their narratives to their most relevant reviewers and funders. As part of the post-grantathon evaluation, it would be important to track mentors’ involvement with their trainees throughout the research projects. This is being planned for the ongoing training in India.

## Conclusion

Our workshop enabled 24 junior investigators to develop and submit research proposals to ICMR for funding, thus attaining its primary goal. Moreover, ICMR ultimately funded all of these proposals. Given this funding commitment and our capacity-building emphasis on evaluation and data collection, these projects may yield important information about the implementation and impact of community-based mental health services throughout India. The grantathon model described here can be modified to build research capacity in other contexts. For example, medical schools could incorporate ‘thesithons’ for planning thesis topics on a common medical problem addressed in different ways by diverse departments. In addition, our capacity-building model could be tested in other LMICs to address the global burden of mental illness. Given that this workshop was not only conceptualised and delivered but also returned results in less than 1 year, the model has the potential to quickly build research capacity and ultimately reduce the mental health treatment gap in resource-limited settings.

## Abbreviations

DMHP: District Mental Health Programme
IMCR: Indian Council for Medical Research
LMIC: Low- and Middle-Income Countries
NMHP: National Mental Health Programme
PGIMER-Dr. RML: Post-Graduate Institute of Medical Education and Research at Dr. Ram Manohar Lohia Hospital
